# Association between dietary total antioxidant capacity and sleep problems and depressive symptoms among U.S. adults

**DOI:** 10.3389/fnut.2024.1450815

**Published:** 2024-09-19

**Authors:** Hong Pan, Shasha Lin

**Affiliations:** ^1^Department of Neurology, Deqing People’s Hospital, Huzhou, China; ^2^Department of Ultrasound, The First Affiliated Hospital of Wenzhou Medical University, Wenzhou, China

**Keywords:** TAC, depressive symptoms, NHANES, sleep problems, sleep duration, trouble sleeping

## Abstract

**Background:**

In this study, we aim to examine the impact of dietary total antioxidant capacity (TAC) on sleep problems and depressive symptoms (DS); besides, we seek to elucidate the potential mediating effect of dietary TAC on the relationships between sleep problems and DS.

**Methods:**

Weighted Kruskal–Wallis tests for continuous variables and Chi-square tests for categorical variables were employed to discriminate between DS and non-DS participants. Multivariable logistic regression and restricted cubic spline analysis were applied to evaluate the associations of TAC with DS and sleep problems.

**Results:**

Among the 21,805 participants, 1,947 participants suffered from DS. Weighted multivariable logistical regression indicated that shorter sleep hours were linked to an increased likelihood of risk of DS even after complete adjustments. Restricted cubic spline cure displayed that TAC was almost non-linearly correlated with DS and sleep problems. Mediation analysis indicated that sleep duration slightly mediated the association between TAC and DS (proportion of mediation: 3.12%, *p* < 0.001).

**Conclusion:**

This study illustrated the inverse association between TAC value and sleep problems and DS. Furthermore, TAC slightly mediated the effect of sleep duration on the DS, and there was a nearly non-linear relationship between TAC and DS, and TAC and sleep problems.

## Introduction

Depression is a prevalent mood disorder characterized by continuous feelings of sadness and a lack of enjoyment in daily activities (1). According to the Global Burden of Disease Study, in 2019, depression was the largest proportion of mental disorders and ranked as the 13th leading cause of disability-adjusted life-years (2). During the COVID-19 pandemic, the overall frequency of depression was 3152.9 instances (2722.5 to 3654.5) per 100,000 people, indicating a rise of 27.6% in 2020 (3). Given the significant impact and burden of depression, it is crucial to identify modifiable factors that can help prevent and mitigate its effects.

Sleep habits play a crucial role in lifestyle, and any deviation from typical sleep patterns can serve as an essential clinical manifestation of depression (4). Individuals with depression often experience sleep disturbances, which are recognized as common symptoms (5). Increasing evidence indicates that sleep disturbances are risk factors for the development and advancement of depressive symptoms (DS) (6, 7).

Dietary total antioxidant capacity (TAC), which refers to the combined and synergistic abilities of antioxidants found in the diet, may offer insights into the body’s ability to combat oxidative stress (8, 9). Previous research has shown that lower levels of antioxidants are associated with an increased likelihood of experiencing depression (10). Moreover, a recent study has confirmed a substantial inverse correlation between total antioxidant capacity in the diet and the likelihood of experiencing depression (11). Additionally, evidence supports the idea that consuming a balanced diet rich in nutrients can be a practical and cost-effective strategy for addressing sleep disturbances (12). For example, vitamin C, a powerful antioxidant, has been shown to protect against sleep deprivation and improve memory function (13). Reduced levels of vitamin C have been associated with shorter sleep duration, diminished sleep quality, and overall poor sleep health (14, 15).

Despite extensive studies conducted on the associations between sleep disturbance and depression, the mechanism linking sleep and depression remains further investigated. In this study, we aim to examine the impact of dietary total antioxidant capacity on both sleep problems and depressive symptoms (DS), and to elucidate the potential mediating effect of dietary TAC on the relationships between sleep problems and DS.

## Methods

### Study population

The NHANES is a continuous national initiative to evaluate the health and nutritional status of participants in the United States (16). Approval for the study protocol has been granted by the Ethics Review Committee of the National Center for Health Statistics (NCHS). Upon recruitment into the survey, all participants sign an informed consent form, ensuring that they are fully aware of the objectives and implications of the study. In the current study, participants were enrolled with complete information on dietary from 2005 to 2018 in NHANES (*n* = 44,279). Next, we excluded participants with missing information on sleep problems (*n* = 14,556), depressive symptoms (*n* = 3,171), and other covariates (*n* = 4,747). Collectively, 21,805 participants were recruited in the final study.

### Estimates of TAC

Dietary intake was evaluated through a 24 h dietary recall (DR) spanning 2 days—day 1 and day 2. Information was gathered using the mobile examination center (MEC) dietary interview and telephone interviews. Dietary TAC was collected from the two-day, 24 h DRs.

The intake of eight antioxidant vitamins (vitamin A, vitamin C, vitamin E, α-carotene, β-carotene, β-cryptoxanthin, lycopene, and lutein-zeaxanthin) were collected in the NHANES dietary interview.

Vitamin C equivalent antioxidant capacity reference values of the above eight antioxidant vitamins were calculated based on the TAC database by Floegel et al. (9). Theoretical TAC was calculated by summing the TAC of the above eight antioxidative vitamins as follows (9):


$$TAC=\sum \;\left[\begin{array}{l} \mathrm{Antioxidant content}\;\left(100\;g/d\right)\\ \times \mathrm{Antioxidant capacity}\;\left(\mathrm{mg}\;VCE/100\;g\right) \end{array}\right]$$

### Definition of DS

DS was tested using the Patient Health Questionnaire (PHQ-9), with a total score of 0–27 points. Those with a total PHQ-9 score of ≥10 were considered to have clinically relevant depression, as the previous research reported (17).

### Sleep measures

Sleep duration was evaluated through the inquiry: “How much sleep do you usually get at night on weekdays or work?” The sleep duration was categorized according to the guidelines provided by the National Sleep Foundation: 6 to 9 h was classified as normal sleep duration, while more than 6 h but less than 9 h was defined as abnormal sleep duration (18). Furthermore, we applied a narrower range of 7 to 8 h for the recommended sleep duration, with abnormal sleep duration defined as less than 7 h or over 8 h, to validate the association between TAC and extreme sleep durations (19).

Trouble sleeping was assessed by the question: “Have you ever told a doctor or other health professional that you have trouble sleeping?” Participants who answered “Yes” were considered to have trouble sleeping.

### Covariates

Covariates, including sociodemographic, economic, lifestyle factors, and chronic medical conditions, were collected.

Sociodemographic factors included age, sex, race, marital status, educational status, and body mass index (BMI). The economic factor was assessed by the ratio of family income to poverty (PIR). Lifestyle factors included smoking status, alcohol status, work activity, and recreational activity.

Chronic medical conditions included stroke, diabetes metallic (DM), hypertension, hyperlipidemia, and coronary heart disease history were collected. The definitions of DM, hypertension, and hyperlipidemia were in accordance with our previous study (20).

### Statistical analyses

Considering the complexity of data in the NHANES, all analyses adopted a method of oversampling, clustering, and stratification to ensure the sample’s representativeness in the US population (13), which were performed with R (version 4.1.2, R Foundation for Statistical Computing, Vienna, Austria).

Continuous variables were presented as weighted means and standard errors (SE), whereas categorical variables were as weighted percentages. Weighted Kruskal–Wallis tests for continuous variables and chi-square tests for categorical variables were employed to discriminate between DS and non-DS participants. TAC were grouped into four quartiles and ln-transformed to obtain an approximate normal distribution. Multivariable logistic regression was applied to investigate the associations of TAC with DS and sleep problems.

## Results

### Characteristics of participants distributed by DS

**Figure 1:**
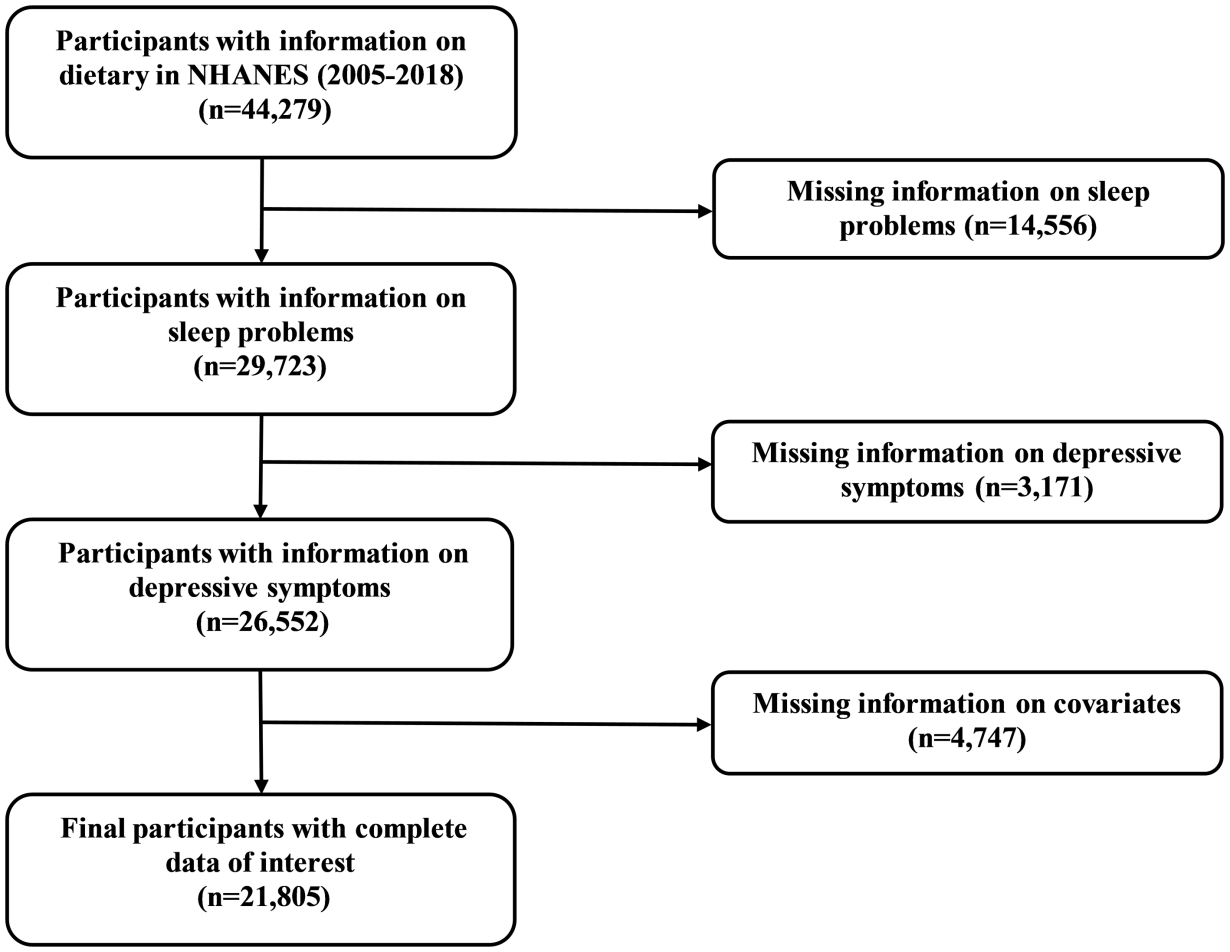
Flow chart of the study population inclusion and exclusion.

Inclusion criteria were illustrated in Figure 1, and 44,279 participants with complete information on dietary were initially enrolled. Following the exclusion criteria, 21,805 participants were ultimately included in the study. Among the 21,805 participants, 1,947 participants were suffering from DS. The baseline characteristics of the participants are detailed in Table 1. Age, sex, BMI, ethnicity, education, marital status, family PIR, alcohol status, smoking status, work activity, recreational activity, stroke, DM, hypertension, hyperlipidemia, coronary heart disease, TAC, and sleep duration were statistically significant between DS and non-DS participants.

**Table 1:** Baseline characteristics of participants with or without depressive symptoms.

Characteristics	Total	Non-DS	DS	*p-*value
Age, (years)	47.39 (0.28)	47.52 (0.30)	45.95 (0.57)	0.0100
Sex, *n* (%)				<0.0001
Female	11,190 (51.32)	9,943 (50.23)	1,247 (63.02)	
Male	10,615 (48.68)	9,915 (49.77)	700 (36.98)	
BMI, (kg/m^2^)				<0.0001
<18.5	308 (1.41)	276 (1.34)	32 (1.40)	
18.5–25	5,730 (26.41)	5,322 (28.46)	408 (23.22)	
25–30	7,126 (32.85)	6,626 (33.25)	500 (26.73)	
≥30	8,641 (39.83)	7,634 (37.50)	1,007 (49.15)	
Race/ethnicity, *n* (%)				<0.0010
Black	4,561 (20.92)	4,151 (10.32)	410 (12.95)	
Mexican	3,033 (13.91)	2,766 (8.07)	267 (8.39)	
White	9,807 (44.98)	8,935 (69.12)	872 (63.34)	
Other Hispanic	2,128 (9.76)	1878 (5.09)	250 (7.80)	
Other race	2,276 (10.44)	2,128 (7.39)	148 (7.52)	
Education, *n* (%)				<0.0001
Less than 12th grade	4,635 (21.26)	3,992 (12.82)	643 (22.34)	
High school	4,970 (22.79)	4,502 (22.17)	468 (27.86)	
Some college	6,700 (30.73)	6,096 (31.91)	604 (35.37)	
College graduate or above	5,500 (25.22)	5,268 (33.10)	232 (14.43)	
Marital status, *n* (%)				<0.0001
Never married	3,910 (17.93)	3,499 (18.50)	411 (23.81)	
Married/living with a partner	13,128 (60.21)	12,249 (64.67)	879 (46.40)	
Widowed/separated /divorced	4,767 (21.83)	4,110 (16.80)	657 (29.79)	
Family PIR				<0.0001
<1.3	6,680 (30.64)	5,665 (19.32)	1,015 (41.20)	
1.3–1.85	2,924 (13.54)	2,626 (10.34)	298 (15.67)	
1.85–3	3,992 (18.13)	3,624 (17.44)	298 (17.84)	
>3	8,279 (38.34)	7,943 (53.43)	336 (26.41)	
Alcohol status, *n* (%)				<0.0001
Never	2,931 (13.44)	2,709 (10.70)	222 (8.60)	
Former	3,460 (15.87)	3,024 (12.23)	436 (18.59)	
Mild	7,682 (35.23)	7,183 (38.39)	499 (28.63)	
Moderate	3,463 (15.88)	3,159 (17.96)	304 (17.96)	
Heavy	4,269 (19.58)	3,783 (20.72)	486 (26.23)	
Smoking status, *n* (%)				<0.0001
Never	12,079 (55.4)	11,296 (57.88)	783 (38.92)	
Former	5,422 (24.87)	4,983 (25.14)	439 (20.59)	
Now	4,304 (19.74)	3,579 (16.98)	725 (40.49)	
Work activity, *n* (%)				0.0200
No	12,567 (57.63)	11,403 (52.37)	1,164 (57.73)	
Moderate	4,881 (22.38)	4,480 (25.30)	401 (22.26)	
Vigorous	855 (3.92)	765 (3.81)	90 (4.40)	
Both	3,502 (16.06)	3,210 (18.52)	292 (15.61)	
Recreational activity, *n* (%)				<0.0001
No	11,024 (50.56)	9,695 (41.96)	1,329 (63.99)	
Moderate	5,867 (26.91)	5,471 (29.33)	396 (23.81)	
Vigorous	1,632 (7.48)	1,552 (8.73)	80 (3.84)	
Both	3,282 (15.05)	3,140 (19.98)	142 (8.36)	
Stroke, *n* (%)				<0.0001
No	20,968 (96.16)	19,177 (97.44)	1791 (93.70)	
Yes	837 (3.84)	681 (2.56)	156 (6.30)	
DM, *n* (%)				<0.0001
No	17,687 (81.11)	16,250 (86.58)	1,437 (81.37)	
Yes	4,118 (18.89)	3,608 (13.42)	510 (18.63)	
Hypertension, *n* (%)				<0.0001
No	12,389 (56.82)	11,438 (63.47)	951 (53.50)	
Yes	9,416 (43.18)	8,420 (36.53)	996 (46.50)	
Hyperlipidemia, *n* (%)				0.0200
No	6,473 (29.69)	6,017 (31.17)	456 (27.24)	
Yes	15,332 (70.31)	13,841 (68.83)	1,491 (72.76)	
Coronary heart disease, *n* (%)				<0.0010
No	20,898 (95.84)	19,079 (96.72)	1819 (94.00)	
Yes	907 (4.16)	779 (3.28)	128 (6.00)	
TAC	8.73 (0.01)	8.76 (0.01)	8.44 (0.04)	<0.0001
Sleep hour, (h)	7.15 (0.02)	7.19 (0.02)	6.77 (0.08)	<0.0001
Sleep hour, *n* (%)				<0.0001
Abnormal	9,688 (44.43)	8,480 (38.91)	1,208 (60.49)	
Normal (6–9 h)	12,117 (55.57)	11,378 (61.09)	739 (39.51)	
Sleep hour, *n* (%)				<0.0001
Abnormal	10,611 (48.66)	9,353 (44.25)	1,258 (64.41)	
Normal (7–8 h)	11,194 (51.34)	10,505 (55.75)	689 (35.59)	
Trouble sleeping, *n* (%)				<0.0001
No	15,958 (73.19)	15,169 (74.75)	789 (37.08)	
Yes	5,847 (26.81)	4,689 (25.25)	1,158 (62.92)	

DS, depressive symptoms; BMI, body mass index; family PIR, family income to poverty; DM, diabetes metallic; TAC, total antioxidant capacity.

### Associations between sleep problems and DS

Interactions between sleep problems and DS were displayed in Table 2. The results indicated that shorter sleep hours were linked to an increased likelihood of risk of DS. The correlation was notable in our initial model (OR = 0.81; 95% CI, 0.75–0.88, *p* < 0.0001) and minimally adjusted model (OR = 0.84; 95% CI, 0.78–0.90, *p* < 0.0001). Even after complete adjustments, the correlation between sleep duration and DS was still significant (OR = 0.86; 95% CI, 0.80–0.92, *p* < 0.0001). We further converted sleep hours to a categorical variable and found that abnormal sleep hours were related to an increased likelihood of DS in all models. In the fully adjusted model, the association between sleep hours and DS remained significant (OR = 1.82; 95% CI, 1.53–2.18, *p* < 0.0001). For trouble sleeping, the association between trouble sleeping and DS remained after adjusting for all variables in model 3 (OR = 4.66; 95% CI, 3.92–5.54, *p* < 0.0001).

**Table 2:** Association of sleep problems with risk of depressive symptoms, NHANES 2005–2018.

	Model 1	*p-*value	Model 2	*p-*value	Model 3	*p-*value
Sleep hour (h)	0.81 (0.75, 0.88)	<0.0001	0.84 (0.78, 0.90)	<0.0001	0.86 (0.80, 0.92)	<0.0001
Sleep hour (6–9 h)						
Normal	Reference		Reference		Reference	
Abnormal	2.40 (2.05, 2.82)	<0.0001	1.97 (1.66, 2.33)	<0.0001	1.82 (1.53, 2.18)	<0.0001
Sleep hour (7–8 h)						
Normal	Reference		Reference		Reference	
Abnormal	2.28 (1.97, 2.64)	<0.0001	1.95 (1.66, 2.28)	<0.0001	1.83 (1.55, 2.16)	<0.0001
Trouble sleeping						
No	Reference		Reference		Reference	
Yes	5.02 (4.34, 5.81)	<0.0001	5.29 (4.49, 6.23)	<0.0001	4.66 (3.92, 5.54)	<0.0001

Model 1: crude model without adjusting. Model 2: adjusted for confounders: age, sex, ethnicity, education, marital status, and PIR. Model 3: adjusted for all confounders.

### Associations between dietary TAC and DS

**Figure 2:**
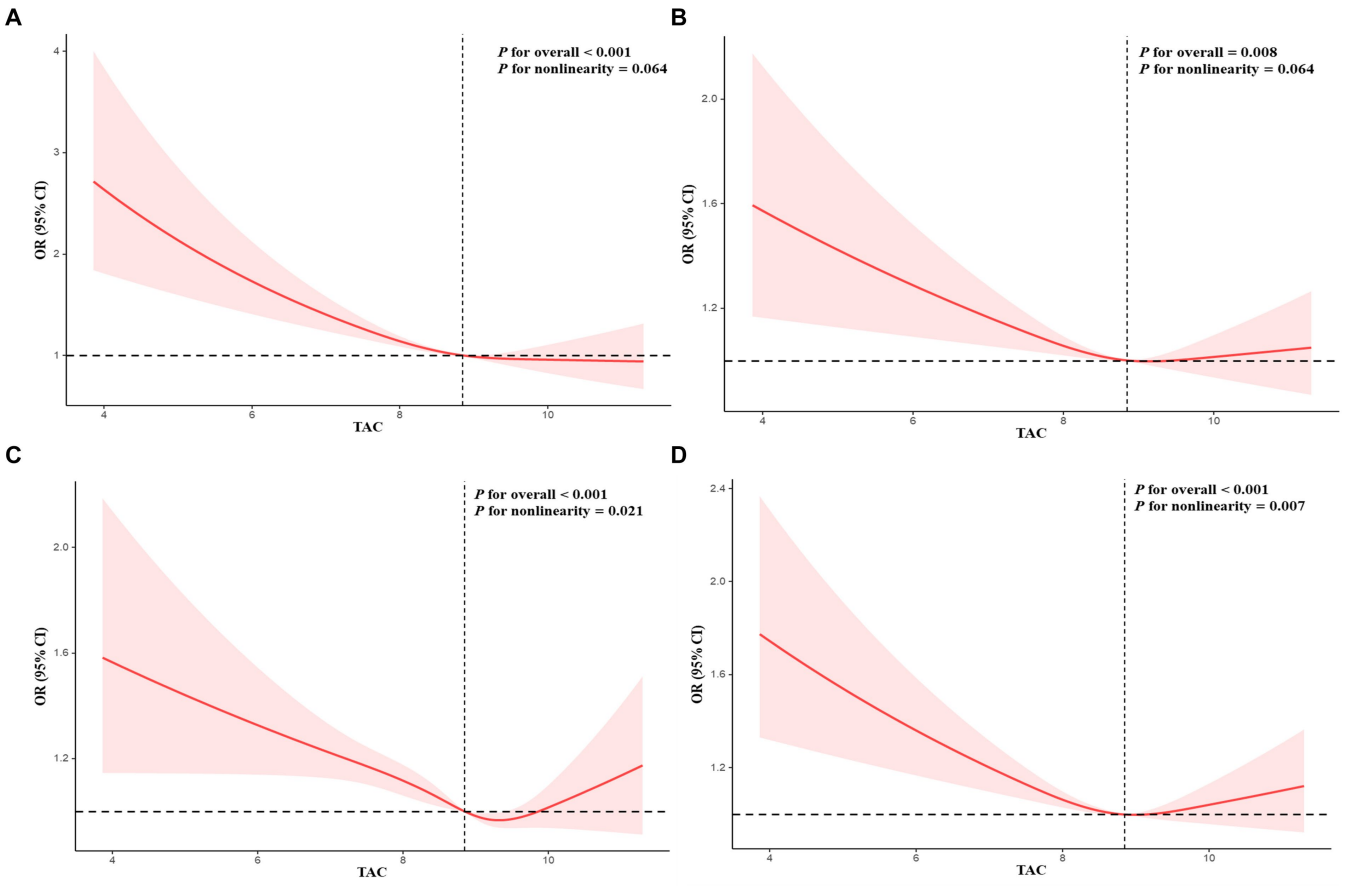
Restricted cubic spline plots of relationships between TAC and depressive symptoms **(A)**, sleep duration (7–8 h as a normal sleep duration) **(B)**, sleep duration (6–9 h as a normal sleep duration) **(C)**, and trouble sleeping **(D)**. Multivariable adjusted ORs (red lines) and 95% CI (pink areas) for risk of abnormal sleep duration, trouble sleeping, and depressive symptoms.

Table 3 presented associations between TAC and DS. The findings in this study revealed that a lower TAC level was linked to a higher probability of DS. The correlation was substantial both in our rudimentary model (OR = 0.70; 95% CI, 0.66–0.76, *p* < 0.0001) and slightly altered model (OR = 0.79; 95% CI, 0.73–0.85, *p* < 0.0001). In the thoroughly adjusted model, the correlation continued to be substantial (OR = 0.86; 95% CI, 0.80–0.93, *p* < 0.0010). As a categorical variable for the TAC, the highest quartile was associated with a significantly reduced risk of DS when compared to the lowest quartile in our crude model (OR = 0.48; 95% CI, 0.39–0.58, *p* < 0.0001) and minimally adjusted model (OR = 0.62; 95% CI, 0.51–0.75, *p* < 0.0001). In the fully adjusted model, the association between the highest quartile of TAC and DS remained positive (OR = 0.77; 95% CI, 0.63–0.94, *p* = 0.0100). Restricted cubic spline cure displayed that the TAC was almost non-linearly correlated with DS (*p* for overall <0.001; *p* for non-linearity = 0.064) (Figure 2A).

**Table 3:** Association of TAC value with risk of depressive symptoms, NHANES 2005–2017.

	Model 1	*p-*value	Model 2	*p-*value	Model 3	*p-*value
TAC	0.70 (0.66, 0.76)	<0.0001	0.79 (0.73, 0.85)	<0.0001	0.86 (0.80, 0.93)	<0.0010
TAC categories						
1st	Reference		Reference		Reference	
2nd	0.65 (0.55, 0.78)	<0.0001	0.76 (0.64, 0.90)	0.0020	0.83 (0.70, 1.00)	0.0500
3rd	0.48 (0.39, 0.58)	<0.0001	0.57 (0.48, 0.69)	<0.0001	0.67 (0.55, 0.81)	<0.0010
4th	0.48 (0.39, 0.58)	<0.0001	0.62 (0.51, 0.75)	<0.0001	0.77 (0.63, 0.94)	0.0100
*p* for trend		<0.0001		0.0050		0.1090

TAC, total antioxidant capacity.

### Associations between dietary TAC and sleep problems

Interactions between TAC values and sleep problems were presented in Table 4. The findings indicated that decreased levels of TAC were linked to an increased likelihood of risk of abnormal sleep duration. This association was significant both in our crude model (OR = 0.85; 95% CI, 0.81–0.89, *p* < 0.0001), minimally adjusted model (OR = 0.90; 95% CI, 0.85–0.94, *p* < 0.0001), and the wholly adjusted model (OR = 0.93; 95% CI, 0.89–0.98, *p* < 0.0010). For the categorical variable of TAC value, the highest quartile had a significantly decreased risk of abnormal sleep duration compared with the lowest quartile in our crude model (OR = 0.69; 95% CI, 0.61–0.77, *p* < 0.0001), minimally adjusted model (OR = 0.78; 95% CI, 0.69–0.88, *p* < 0.0010), and completely adjusted model (OR = 0.84; 95% CI, 0.74–0.96, *p* = 0.0100). The trend for *p*-value remained significant in all models (*p* < 0.05). For trouble sleeping, the association between the highest quartile of TAC value and risk of trouble sleeping vanished after adjusting for all variables in model 3 (OR = 0.95; 95% CI, 0.89–1.01, *p* = 0.0900). The trend for *p-*value was insignificant in all models (*p* > 0.05). Restricted cubic spline cure displayed that TAC value was non-linearly correlated with sleep duration (*p* for overall <0.001; *p* for non-linearity = 0.021) (Figure 2C) and trouble sleeping (*p* for overall <0.001; *p* for non-linearity = 0.007) (Figure 2D).

**Table 4:** Associations of TAC value with sleep problems, NHANES 2005–2018.

	Model 1	*p-*value	Model 2	*p-*value	Model 3	*p-*value
Sleep hour (6–9 h)						
TAC	0.85 (0.81, 0.89)	<0.0001	0.90 (0.85, 0.94)	<0.0001	0.93 (0.89, 0.98)	<0.0001
TAC categories						
1st	Reference		Reference		Reference	
2nd	0.76 (0.68, 0.85)	<0.0001	0.83 (0.74, 0.93)	0.0020	0.86 (0.76, 0.97)	0.0100
3rd	0.70 (0.63, 0.79)	<0.0001	0.79 (0.70, 0.90)	<0.0010	0.84 (0.75, 0.96)	0.0100
4th	0.69 (0.61, 0.77)	<0.0001	0.78 (0.69, 0.88)	<0.0010	0.84 (0.74, 0.96)	0.0100
*p* for trend		<0.0001		0.0030		0.0240
Sleep hour (7–8 h)						
TAC	0.86 (0.82, 0.89)	<0.0001	0.89 (0.85, 0.93)	<0.0001	0.92 (0.88, 0.96)	<0.001
TAC categories						
1st	Reference		Reference		Reference	
2nd	0.80 (0.71, 0.89)	<0.0001	0.85 (0.75, 0.95)	0.0100	0.87 (0.78, 0.98)	0.0200
3rd	0.73 (0.65, 0.83)	<0.0001	0.80 (0.71, 0.91)	<0.0010	0.85 (0.75, 0.96)	0.0100
4th	0.69 (0.62, 0.77)	<0.0001	0.76 (0.67, 0.85)	<0.0001	0.81 (0.72, 0.92)	<0.0010
*p* for trend		<0.0001		0.0040		0.0220
Trouble sleeping						
TAC	0.90 (0.85, 0.94)	<0.0001	0.91 (0.86, 0.97)	0.002	0.95 (0.89, 1.01)	0.0900
TAC categories						
1st	Reference		Reference		Reference	
2nd	0.88 (0.76, 1.02)	0.1000	0.87 (0.75, 1.01)	0.0700	0.90 (0.77, 1.06)	0.1900
3rd	0.76 (0.67, 0.87)	<0.0001	0.75 (0.66, 0.87)	<0.0010	0.80 (0.69, 0.93)	0.0040
4th	0.82 (0.72, 0.93)	<0.0020	0.85 (0.74, 0.98)	0.0300	0.94 (0.82, 1.09)	0.4100
*p* for trend		0.1920		0.2400		0.5440

TAC, total antioxidant capacity.

### Mediation and sensitivity analysis

**Figure 3:**
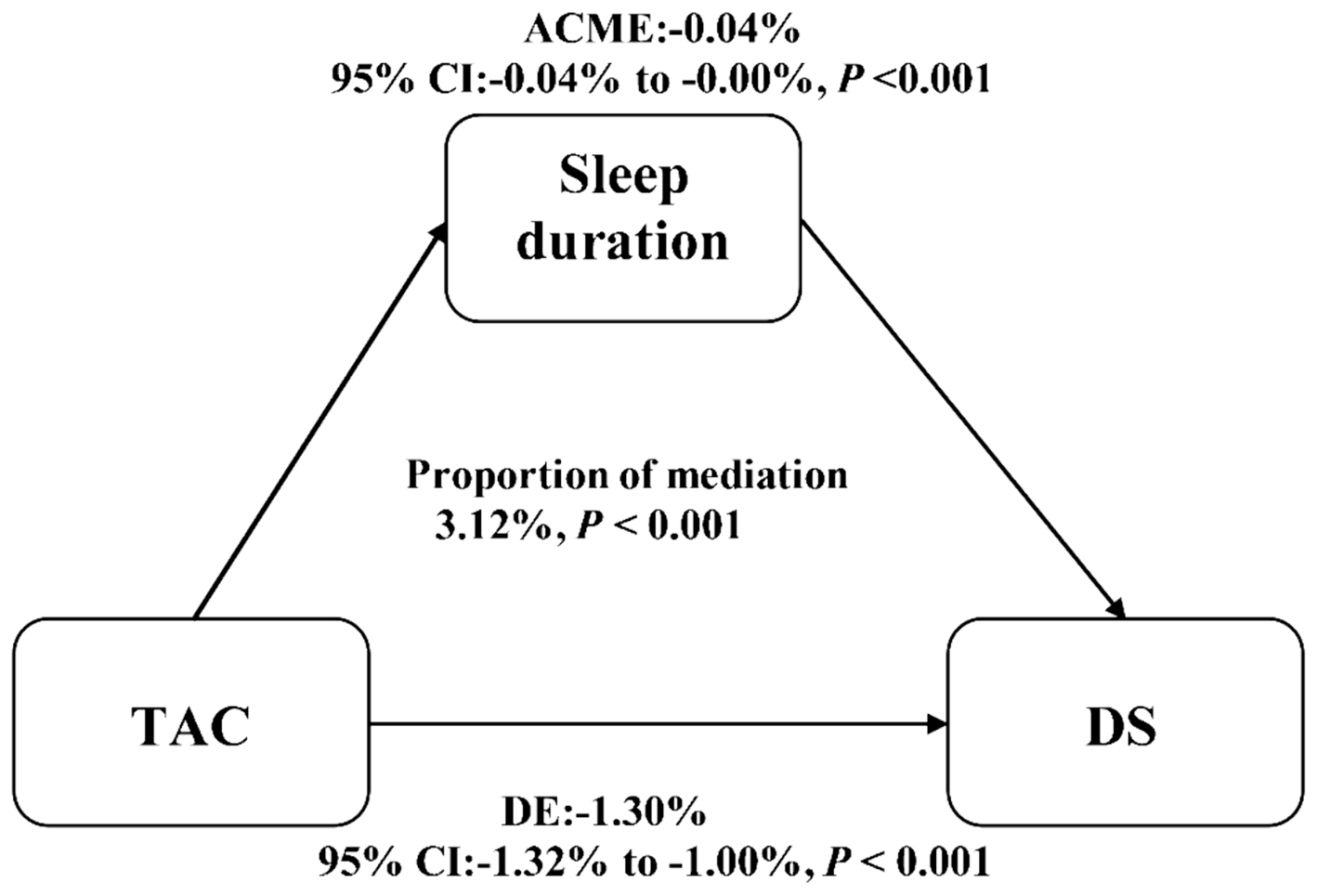
Estimated proportion of the association between TAC and DS mediated by sleep duration. Models were adjusted for all confounders. TAC, total antioxidant capacity. DS, depressive symptoms; IE, the estimate of the indirect effect; DE, the estimate of the direct effect; proportion of mediation = IE/DE + IE.

Mediation analysis was displayed in Figure 3. Sleep duration (as a continuous variable) slightly mediated the association between TAC (as a categorical variable) and DS (proportion of mediation: 3.12%, *p* < 0.001). In addition, sensitivity analysis was conducted to explore the stability between sleep duration (7–8 h as a normal sleep duration), TAC and DS. Results showed sleep duration (7–8 h as a normal sleep duration) was correlated with TAC and DS (Tables 2, 4). Restricted cubic spline cure displayed that TAC was positively correlated with sleep duration (7–8 h as a normal sleep duration) (*p* for overall = 0.008; *p* for non-linearity = 0.064) (Figure 2B).

## Discussion

To our knowledge, this study elucidated the association between TAC, sleep problems, and DS for the first time. In this study, it illustrated an inverse association between TAC and sleep problems, TAC and DS, and sleep problems and DS. Furthermore, TAC slightly mediated the effect of sleep duration on the DS, and there was a nearly non-linear relationship between TAC and DS, and TAC and sleep problems.

Several studies concentrated on the association between TAC, depression, and sleep disorders were conducted, whereas the results were inconsistent. A study combining 400 postmenopausal women indicated an association between oxygen radical absorbance capacity (ORAC) assessing TAC and sleep problems but not depressive mood (21). In addition, another study combining 911 men and women also confirmed the absence of an association between ORAC and DS (22). However, another small sample study combining 175 postmenopausal women illustrated that there was an inverse association between ORAC and depression (23). A larger sample combining 3,297 healthy adults demonstrated that ferric reducing ability of plasma (FRAP) was linked to a lower chance of suffering depression (11). Another study combining 265 type 2 diabetic patients indicated that higher tertile of FRAP and ORAC was associated with decreased chances of sleeping poorly and DS (24). The heterogeneity of these conclusions might be due to the relatively small sample and the difference in the sample population.

This study confirmed a negative relationship between TAC and sleep problems. An experimental study using models of flies and mice illustrated that sleep deprivation could cause the accumulation of reactive oxygen species (ROS) in the gut and consequently be accompanied by oxidative stress (25). Another animal study in drosophila found that short-sleeping drosophila mutants were more prone to suffer from acute oxidative stress, and overexpression of antioxidant genes in wide-type flies could influence the amount of sleep (26). A cross-sectional study including 6,300 participants from NHANES demonstrated that the Oxidative Balance Score, including five pro-oxidants and 15 antioxidants, was negatively correlated with sleep disorder and positively linked to sleep duration (27).

This study illustrated a significant relationship between TAC and DS. Depression is a complex mental health disorder, the exact mechanism of which is not fully understood. One of the mechanisms resulting in depression is called the “mitochondria theory of depression” (28). In patients with major depressive disorder, substance correlated with mitochondrial fragmentation was increased (29). Furthermore, the intensity of depressive symptoms correlates with alterations in protein concentrations linked to mitochondrial dynamics and mitophagy (29). Disrupted mitochondria could release excess products, inhibit neurotransmitter release, and decrease synaptic activity, ultimately damaging the brain and increasing the risk of depressive disorders (30). Dietary intake, a modifiable lifestyle behavior, has aroused growing interest in the treatment of depression in recent years (31). Two meta-analyses confirmed that dietary vitamin A, vitamin C, vitamin E, and beta-carotene intake are inversely associated with depression (32, 33). In addition, a study including 17,401 adults from NHANES indicated that dietary intake, such as alpha-carotene and beta-carotene, are inversely correlated with the risk of depressive symptoms (34). Consistent with the above studies, this study also illustrated an inverse relationship between TAC and the higher odds of DS.

The novelty and strengths of this study may be as follows: firstly, this study utilized a large sample size from a well-established nationwide cohort to demonstrate the relationship between TAC, sleep problems, and DS and implies that intake of food rich in antioxidant capacity may be a method to decrease the frequency of sleep problems and DS. Secondly, this study utilized RCS curves to explore the correlation between TAC, sleep problems, and DS. RCS curves show that within a specific range, sleep abnormalities and the risk of DS decrease virtually in a straight line as the TAC increases. However, beyond this range, the decreasing trend becomes almost nonexistent. The results indicated that excessive intake of oxidants may aggravate sleep problems and DS. Further research may focus on the specific dose of oxidants to decrease the frequency of sleep problems and DS significantly. Thirdly, this study indicated that only 3.12% of the association between TAC and the risk of DS was explained by sleep duration, the exact mechanisms between TAC and sleep problems, TAC, and DS need to be further investigated.

However, this study had several limitations. Although we made efforts to account for potential confounding factors, it was not possible to eliminate the potential influence of other covariates in this study. Additionally, the generalizability of our findings to other populations may be limited as the sample used in this research was obtained from the US database. Lastly, the definition of trouble sleeping is relatively subjective and lacks an objective score.

## Conclusion

This study illustrated the inverse association between TAC and sleep problems, TAC and DS, and sleep problems and DS. Furthermore, TAC slightly mediated the effect of sleep duration on the DS, and there was a nearly non-linear relationship between TAC and DS, and TAC and sleep problems.

## Data Availability

All data are available at NHANES website https://www.cdc.gov/nchs/nhanes/index.htm.
